# Comprehensive Analysis of the ARF Gene Family Reveals Their Roles in Chinese Chestnut (*Castanea mollissima*) Seed Kernel Development

**DOI:** 10.3390/biology14101460

**Published:** 2025-10-21

**Authors:** Xili Liu, Yun Li, Manman Liang, Dongsheng Wang, Meng Wang, Yi Lu, Xia Liu, Haie Zhang, Xiangyu Wang, Liyang Yu

**Affiliations:** 1 Engineering Research Center of Chestnut Industry Technology, Ministry of Education, Hebei Normal University of Science and Technology, Qinhuangdao 066004, China; liuxili15753733443@163.com (X.L.); wdsgfly@126.com (D.W.); 13223362010@163.com (M.W.); luy2021@126.com (Y.L.); zhang33haie4@163.com (H.Z.); 2 Research Center for Rural Vitalization, Hebei Normal University of Science and Technology, Qinhuangdao 066004, China; liyun0118@126.com; 3 College of Horticulture Science and Technology, Hebei Normal University of Science and Technology, Qinhuangdao 066004, China; liangmm2023@163.com; 4 College of Smart Agriculture, Chongqing University of Arts and Sciences, Chongqing 402160, China; liuxiavip8@163.com; 5 The Office of Scientific Research, Hebei Normal University of Science and Technology, Qinhuangdao 066004, China; 6 Hebei Yanshan Special Industrial Technology Research Institute, Qinhuangdao 066004, China

**Keywords:** ARF gene family, *Castanea mollissima*, developmental stage, RNA-seq, seed size, starch content, enzyme activity

## Abstract

**Simple Summary:**

Yield and quality are the primary objectives in *Castanea mollissima* breeding. The kernel of *C. mollissima* primarily accumulates starch as its main storage substance, and the starch content is a key factor determining kernel quality and yield. The auxin response factor (ARF) gene family plays critical roles in plant growth, development, and nutrient accumulation. In this study, 18 ARF family members were identified in the *C. mollissima* genome and subjected to systematic bioinformatic analysis. By examining morphological traits, starch content, and amylase activity at five post-anthesis stages of kernel development, combined with correlation analysis and weighted gene co-expression network analysis (WGCNA), we revealed that specific *ARF* genes are crucial for coordinating seed kernel development and starch accumulation. Notably, we discovered that *CmARF5a* and *CmARF18* may act as key repressors of starch accumulation. Key *ARF* genes potentially regulating starch metabolism were further screened, and their dynamic expression patterns during seed kernel development were validated via RT-qPCR. This study provides valuable insights into the molecular mechanisms underlying starch accumulation in *C. mollissima* and functional characterization of *ARF* genes.

**Abstract:**

Auxin response factors (ARFs) are a class of significant transcription factors that play crucial roles in the regulation of plant growth and development. Although *ARF* genes have been extensively characterized in various plants, their functions in perennial woody crops, particularly their involvement in regulating starch accumulation—a key determinant of yield and quality in *Castanea mollissima*—remain largely unexplored. To address this knowledge gap, we conducted a comprehensive study of the ARF gene family in the *C. mollissima*. In this study, 18 *CmARF* members, exhibiting diverse physicochemical properties, were identified within the *C. mollissima* genome. These *CmARFs* were categorized into four groups. Dispersed duplication emerged as the primary mechanism driving the expansion of the *CmARF* gene family. As *C. mollissima* seed kernels developed, notable changes were observed in starch content and the activity of enzymes related to starch biosynthesis, particularly a significant decrease in GBSS activity, which corresponded with an increase in seed kernel size and starch content. Transcriptome analysis delineated the expression patterns of *CmARF* genes during the development of *C. mollissima* seed kernels. A key novel finding of our research is that *CmARF5a* and *CmARF18* are hypothesized to act as pivotal repressors of starch accumulation. This hypothesis is based on their expression profiles, strong negative correlations with physiological indicators, and WGCNA. Notably, the lack of correlation between these *CmARFs* and the expression of core starch biosynthetic genes suggests a potential indirect regulatory mechanism, offering a new perspective on ARF function in storage organ development. This study not only provides the first comprehensive characterization of the CmARF family but also offers a theoretical framework and candidate genes for future functional research on *C. mollissima* seed kernel development and starch biosynthesis.

## 1. Introduction

Transcription factors (TFs) are proteins that play a pivotal role in regulating gene expression by either promoting or inhibiting transcription through their interaction with DNA sequences near specific genes [1]. Among these, auxin response factors (ARFs) are crucial TFs in plants [2]. An ARF typically comprises three conserved regions: the B3 DNA-binding domain (DBD) at the N-terminus, the C-terminal domain (CTD) featuring Phox and Bem1p (PB1) dimerization regions, and an intervening variable middle region (MR) [3]. The DBD is characterized by its specific DNA-binding activity, enabling it to regulate gene expression by binding to the auxin response element (AuxRE) within the target gene sequence TGTCTC [4,5]. The MR, positioned between the DBD and CTD, varies in composition and is categorized based on its transcriptional activation or inhibition activity, dependent on the amino acid content. The CTD, a relatively conserved domain, facilitates the interaction of Aux/IAA proteins with ARF domain III/IV through motifs such as Phe-Gly-Arg (FGR) and Ser-Thr/Ser-Ser (STS), or the interaction between ARF proteins themselves, thereby modulating the expression of genes responsive to auxin [6,7]. Dimerization within these regions is essential for the accurate regulation of gene transcription and the integration of various signaling pathways mediated by ARF proteins [7].

To date, *ARF* genes have been identified and characterized in a multitude of plant species, including *Arabidopsis thaliana* (23) [4], *Oryza sativa* (25) [8], *Zea mays* (31) [9], *Ipomoea batatas* (29) [10], *Malus domestica* (31) [11], *Vitis vinifera* (19) [12], *Prunus mume* (17) [13], *Prunus persica* (17) [14], and *Cajanus cajan* (17) [15]. Extensive research has explored the diverse biological functions of *ARF* genes in plant growth and development, as well as their responses to environmental stresses. In *A. thaliana*, specific *ARFs* such as *AtARF5*, *AtARF6*, *AtARF7*, *AtARF8*, and *AtARF19* have been implicated in critical growth and developmental processes, including embryonic axis elongation, floral organ development, lateral root formation, and fruit development [16,17,18,19,20,21,22]. Notably, *AtARF6* and *AtARF8* are involved in the regulation of petal and stamen development through the induction of jasmonic acid synthesis, which subsequently activates the expression of R2R3-type MYB TFs *MYB21* and *MYB24*, promoting cell elongation in petals and stamens [19]. Additionally, *AtARF7* and *AtARF19* play a significant role in lateral root formation by directly activating the *LBD*/*ASL* gene [20]. The *LBD*/*ASL* serves as a downstream effector in auxin signaling, with its functional loss or dominant inhibition resulting in defects in lateral root development [20]. *OsARF6* and *OsARF17* can bind to the ILA1 promoter and exert control over *O. sativa* leaf angle by regulating the biosynthesis of secondary cell walls at leaf junctions [23]. Additionally, *OsARF6* is involved in the miR167a-OsARF6-OsAUX3 signaling pathway, which plays a critical role in determining the grain length and weight in *O. sativa* [24]. In *P. mume*, *PmARF13* and *PmARF17* are implicated in developmental processes and functional expression [13]. In *M. domestica*, *MdARF13* influences anthocyanin biosynthesis through the Aux/IAA-ARF signaling pathway [25]. In *Pinus koraiensis*, two genes, *Pkor01G00962.1* and *Pkor07G00704.1*, have been identified as central components of the PkorARF gene family, crucially regulating embryonic development [26]. Moreover, *ARF* genes also play a significant role in plant responses to environmental stress. For instance, *OsARF6* and *OsARF19* mitigate oxidative damage induced by salt stress by activating genes for antioxidant enzymes such as superoxide dismutase (SOD) and peroxidase (POD), and interact with the abscisic acid (ABA) signaling pathway to regulate the synthesis of osmoregulatory substances like proline and soluble sugars [27]. The complexity of the auxin response in plants highlights the ARF gene family as a pivotal element in the auxin signaling pathway, essential for elucidating the regulatory mechanisms underlying plant growth and development [2].

Despite extensive studies on *ARF* genes across numerous species, revealing their diverse roles in growth, development, and stress responses, their functions in perennial woody crops remain comparatively less explored. This is particularly true for their potential role in regulating economically critical traits such as starch accumulation. *C. mollissima* is a deciduous tree of significant agronomic value, whose seed kernels are a vital food source in the Northern Hemisphere due to their high starch content alongside minerals and vitamins [28,29,30]. However, while the ARF gene family has been systematically characterized in many herbaceous and woody plants, a comprehensive analysis in *C. mollissima* is still lacking. More specifically, a critical knowledge gap exists regarding whether *CmARF* genes participate in the regulation of starch biosynthesis during seed kernel development—a fundamental process that determines the yield and quality of this perennial crop. Elucidating this link is essential for advancing our understanding of carbon allocation in woody perennials and provides potential genetic targets for breeding improvement.

Therefore, this study aimed to identify and systematically characterize the *ARF* genes in the *C. mollissima* genome, including analyses of their physicochemical properties, gene structure, phylogenetic evolution, *cis*-acting elements, and duplication events. Furthermore, by measuring dynamic changes in seed kernel size, starch content, and the activity of starch biosynthesis-related enzymes across five developmental stages, and integrating these data with transcriptome profiles, we sought to decipher the molecular mechanisms governing seed kernel development. Our correlation analyses and WGCNA results suggest that *CmARF5a* and *CmARF18* may play significant roles in the development of *C. mollissima* seed kernels and in starch biosynthesis. These findings provide crucial theoretical insights into the role of *CmARF* genes in seed kernel development and establish a foundation for future functional studies.

## 2. Materials and Methods

### 2.1. Identification and Phylogenetic Analysis of CmARFs

The genome data for *Castanea mollissima* was obtained from the Castanea Genome Database (http://castaneadb.net/#/) (accessed on 7 January 2025). Hidden Markov Model (HMM) files pertaining to ARF proteins were sourced from the Pfam database (http://pfam-legacy.xfam.org/) (accessed on 10 January 2025). Reference sequences of AtARF proteins were downloaded from the TAIR database and utilized to conduct a BLAST v.2.2.30+ search against the complete protein sequence database of *C. mollissima*. HMMER 3.0 facilitated the analysis of proteins selected in the preceding step to isolate candidate proteins. Validation of the ARF conserved domain in CmARF proteins was performed using NCBI-CDD and SMART. The physicochemical properties, subcellular localization, and motif arrangements of all CmARF proteins were predicted using tools such as Expasy, WoLF-PSORT, and MEME, with default parameters. The gene structure of CmARF, based on exon-intron positioning, was visualized using TBtools v. 2.313, employing data from gff3 files [31]. A phylogenetic tree, which included ARF proteins from both *C. mollissima* and *A. thaliana*, was constructed using MEGA 7.0 with the maximum likelihood method (Table S1). Secondary and three-dimensional structures of CmARF proteins were predicted using SOPMA and SWISS-MODEL, respectively.

### 2.2. Chromosomal Distribution and Collinear Analysis of CmARFs

Chromosomal locations of the *CmARF* genes were extracted from the *C. mollissima* gff3 file and visualized using TBtools v. 2.313 [31]. Genomic data for *A. thaliana*, *Q. robur*, *S. lycopersicum*, *V. vinifera*, *Z. mays*, *O. sativa*, and *P. bretschneideri* were downloaded from the Phytozome database (https://phytozome-next.jgi.doe.gov/) (accessed on 3 March 2025). Collinear among these species and *C. mollissima* was analyzed using MCScanX v1.0 [32]. Non-synonymous (Ka) and synonymous (Ks) substitution rates of homologous genes within homologous fragments were calculated using the “add_ka_and_ks_to_collinearity” function in MCScanX v1.0. Dot-plots illustrating homologous collinear within the *C. mollissima* genome were generated using TBtools v. 2.313, and the median Ks values were calculated using a custom script [33]. Duplication types of *CmARFs* were identified using the “duplicate_gene_classifier” in MCScanX v1.0, and WGD or segmental duplications were differentiated using a previously described method [34].

### 2.3. Cis-Acting Elements, GO/KEGG Enrichment, Transcription Factors (TFs) Regulatory and Protein-Protein Interaction (PPI) Networks Analysis

The upstream 2000 bp sequences of the *CmARF* genes were uploaded to Plant-CARE to predict *cis*-acting elements [35]. TBtools v. 2.313 facilitated the GO and KEGG enrichment analyses [36]. The regulatory roles of TFs within the 2000 bp upstream regions of the *CmARF* genes were inferred using the Plant Transcriptional Regulatory Map (PTRM). Visualization of these predictions was accomplished using Cytoscape software (v3.9.1) [37]. The potential interactions between CmARF proteins were predicted using the STRING database (https://cn.string-db.org/) (accessed on 13 March 2025) [38].

### 2.4. Plant Materials and Phenotypic Determination

The cultivar ‘Xinglong No. 4’ of *C. mollissima* has demonstrated notable agronomic characteristics, particularly due to its high starch content in the kernels. These were observed at the experimental base of Hebei Normal University of Science and Technology, located in Qinhuangdao City, Hebei Province (39°66′ N, 119°22′ E). Samples from ‘Xinglong No. 4’ *C. mollissima* were harvested at 60, 70, 80, 90, and 100 days (S1 to S5) post-flowering. The mass and dimensions of the kernels—longitudinal, transverse, and lateral diameters—were measured using an analytical balance and a vernier caliper, respectively. Amylose content was determined using the Amylose Content Assay Kit (Cominbio, Suzhou, China) based on an enzymatic method. In this assay, amylose was separated and hydrolyzed to glucose, which was then quantified using the GOPOD reagent at 505 nm. The kit-provided standard (a defined amylose solution) was used for calibration. Amylopectin content was quantified using the Amylopectin Content Assay Kit (Cominbio, Suzhou, China) based on the dual-wavelength method (measuring absorbance at 550 nm and 743 nm). The calculation was performed using the standard curve equation (y = 0.0607x + 0.0076) provided and validated by the kit manufacturer, which is established using purified amylopectin standards. Enzyme activities for adenosine diphosphate glucose pyrophosphorylase (AGPase), soluble starch synthase (SSS), granule-bound starch synthase (GBSS), and starch branching enzyme (SBE) were assessed using a kit provided by Keming Biotechnology Co., Ltd., Suzhou, China [39].

### 2.5. RNA-Sequencing, DEG and WGCNA, RT-qPCR Validation

The RNAprep Pure Plant Kit (Tiengen, Beijing, China) was utilized for RNA extraction from *C. mollissima* kernels. RNA purity was evaluated using the Agilent 2100 Bioanalyzer (Agilent, San Diego, CA, USA). Subsequently, the NEBNext^®^ Ultra™ RNA Library Prep Kit for Illumina (San Diego, CA, USA) facilitated the construction of cDNA libraries. Sequencing was performed on the NovaSeq 6000 system (Illumina, San Diego, CA, USA), and the RNA-seq data have been deposited in the NCBI database (BioProject number: PRJNA1268182). Hisat2 v2.0.5 aligned the clean data to the reference genome, specifically the published N-11 *C. mollissima* genome, which was obtained from the Castanea genome database [40]. FeatureCounts and DESeq2 v1.4.5 were employed to calculate the FPKM (Fragments Per Kilobase of transcript per Million mapped reads) of genes and to identify DEGs [41,42]. WGCNA was conducted using WGCNAshiny in TBtools v. 2.313 [31]. Pearson correlation analysis identified modules significantly correlated with the physiological and biological traits of *C. mollissima* seed kernels of ‘Xinglong No. 4’ at various developmental stages. RT-qPCR analysis was performed using RNA isolated from seed kernels at five developmental stages to validate the temporal expression patterns of selected *CmARF* genes. The PrimeScript RT Master Mix (Takara Biotechnology Co., Dalian, China) was used for the reverse transcription of total RNA. Primer Premier designed specific primers for the CmARF genes [43]. The ABI 7500 Real-Time PCR system (Applied Biosystems Inc., Foster City, CA, USA) conducted RT-qPCR experiments using TB Green Premix Ex Taq (Takara). As in our previous extensive research [28,44,45,46,47,48], the *18S* gene was selected as a reference gene due to its relatively stable expression levels in various cell types and physiological conditions of *C. mollissima*. Additionally, the 2^−ΔΔCt^ method was used to analyze the relative gene expression levels. Information about the primers is available in Table S2.

### 2.6. Statistical Analysis

All experiments were performed with three independent biological replicates. Data are presented as the mean ± standard deviation (SD). Statistical comparisons were conducted by one-way analysis of variance (ANOVA) followed by Tukey’s post hoc test. Differences were considered statistically significant at a *p*-value < 0.05. All statistical analyses were performed using GraphPad Prism 9.5. Additionally, Pearson correlation analysis was performed using IBM SPSS Statistics 26 to assess: (1) the relationships between CmARF genes expressions (FPKM) and phenotypic indicators, and (2) the correlation between RNA-seq (FPKM) and RT-qPCR results for CmARF genes. A *p*-value < 0.05 was considered statistically significant.

## 3. Results

### 3.1. Identification and Phylogenetic Analysis of the CmARFs

**Figure 1:**
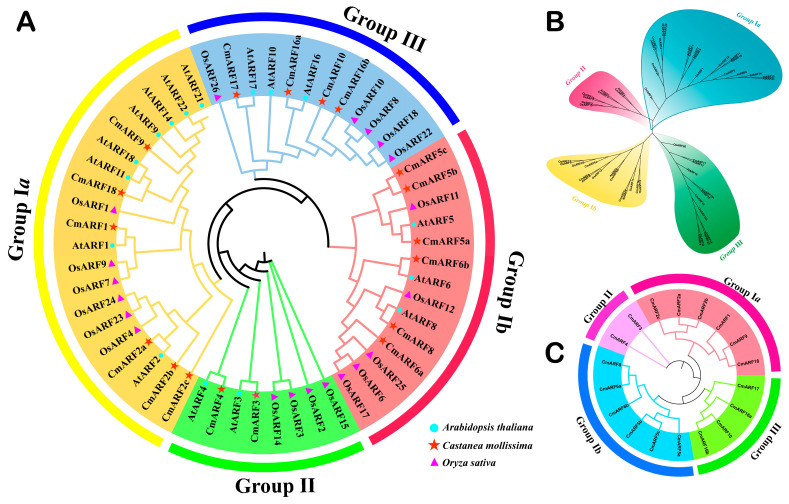
The phylogenetic tree of *ARF* genes of *C. mollissima*, *A. thaliana*, and *O. sativa*. (**A**) Rooted phylogenetic tree of *ARF* genes of *C. mollissima*, *A. thaliana*, and *O. sativa*. (**B**) Unrooted phylogenetic tree of *ARF* genes of *C. mollissima*, *A. thaliana*, and *O. sativa*. (**C**) Rooted phylogenetic tree of *ARF* genes of *C. mollissima*. MEGA 7.0 was used to construct the phylogenetic tree based on the protein sequences with maximum likelihood method.

In the genome of *C. mollissima*, a total of 18 *ARF* genes were identified based on sequence similarity and the presence of conserved domains (Table S3). The molecular weights of the CmARFs range from 63.15 kDa (CmARF17) to 119.12 kDa (CmARF5b). Their GRAVY values span from −0.669 (CmARF2a) to −0.249 (CmARF16b), confirming the hydrophilic nature of CmARF proteins. The theoretical isoelectric points (pI) of these proteins vary from 5.26 (CmARF5a) to 8.71 (CmARF6a), with only CmARF10 and CmARF6a possessing pI above seven, indicating that the majority of CmARF proteins are acidic (pI < 7). The aliphatic index ranges from 64.04 (CmARF2a) to 77.00 (CmARF6a), while the instability index ranges from 45.51 (CmARF10) to 66.25 (CmARF5c), suggesting that these proteins are generally unstable. Predictions of subcellular localization reveal that while CmARF6b is localized to both the nucleus and cytoplasm, other members are exclusively nuclear. Phylogenetic analysis comparing *ARF* genes from *C. mollissima*, *A. thaliana*, and *O. sativa* grouped the *CmARF* genes into four distinct clusters: Group Ia and Ib each contain six *CmARF* members; Group III includes four; and Group II comprises just two, *CmARF3* and *CmARF4* (Figure 1). In the cross-species phylogenetic tree, as expected from their closer evolutionary relationship as dicots, all *CmARF* members, except *CmARF5b*, *CmARF5c*, *CmARF10*, and *CmARF16b*, exhibit closer genetic relationships with *AtARF* members than with *OsARF* members.

### 3.2. Characterization of the CmARFs

**Figure 2:**
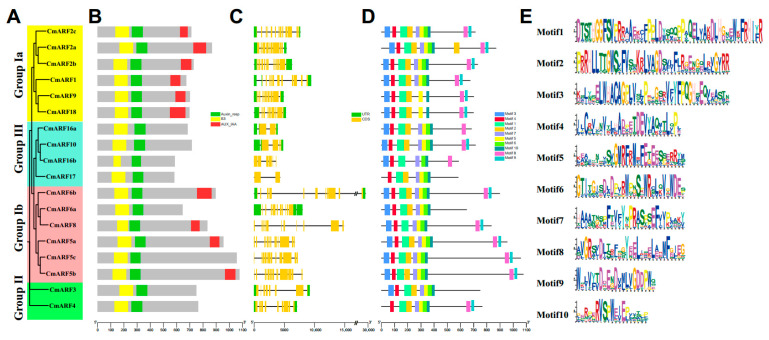
The conserved domains, gene structure, and conserved motifs distribution of *CmARF* genes. (**A**) Phylogenetic analysis of *CmARF* genes. (**B**) The conserved domains of *CmARF* genes. (**C**) Gene structure of *CmARF* genes. (**D**) The conserved motifs of CmARF proteins. (**E**) The sequence of ten conserved motifs in CmARF proteins.

Each of the CmARF proteins incorporates B3 DBDs and ARF. Notably, ARF members in Groups II and III, along with CmARF6a and CmARF5c from Group Ib, lack C-terminal AUX/IAA domains (CTD), whereas other CmARF members possess the complete ARF domain architecture (DBD-ARF-CTD) (Figure 2A,B). The exon-intron structures of CmARF members vary among groups (Figure 2C): those in Groups Ia and Ib typically contain 13–15 introns, whereas members in Group III have between 2 and 8 introns. *CmARF3* and *CmARF4* from Group II contain 10 and 11 introns, respectively. These structural features align with the phylogenetic relationships previously described (Figure 1). Motifs 1, 4, and 10 were identified in all CmARF members, underscoring their significant roles within the gene family (Figure 2D). Additionally, some members display a motif arrangement that deviates from others within the same group but matches that of members in different groups. For instance, CmARF6a and CmARF3, belonging to Groups Ib and II, respectively, share identical motif orders, differing from other members within their respective groups. This suggests evolutionary changes in non-motif sequences among some CmARF members, leading to the retention of identical motif orders across different groups.

### 3.3. Chromosomal Location and Duplication Type Analysis of CmARFs

**Figure 3:**
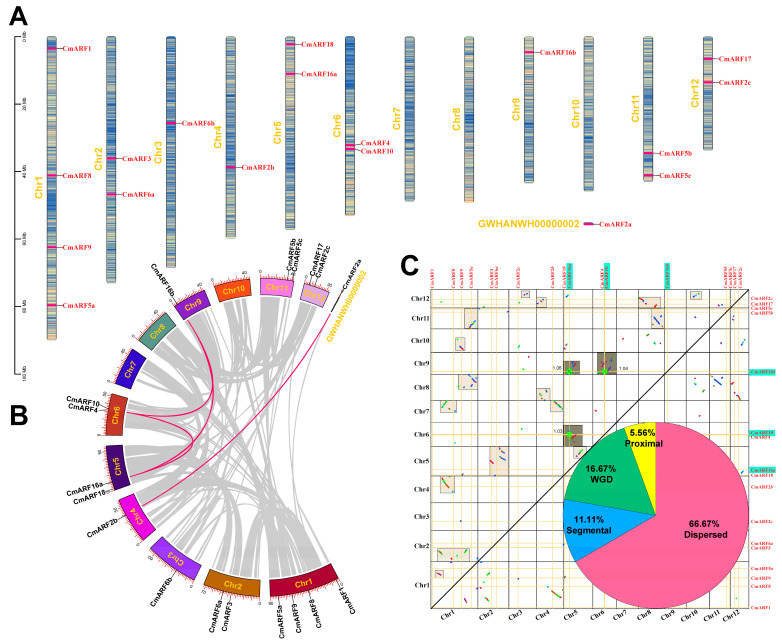
Chromosome distribution and duplication type analysis of *CmARF* genes. (**A**) Chromosome distribution of *CmARF* genes. The color of segments in the chromosomes shows the gene density of the corresponding region. (**B**) Collinear relationship within *C. mollissima* genome. (**C**) Homologous collinear dot-plot within *C. mollissima* genome. The collinear blocks from WGD containing *CmARF* genes are marked in the gray boxes of the figure, and the blocks length and median Ks of the collinear blocks are marked. The genes highlighted in green are identified as from WGD events. The pie chart in the bottom right corner of the dot-plot shows the proportion of *CmARFs* for different duplication types.

The *C. mollissima* genome encodes 17 *CmARF* genes situated on various chromosomes, with the exception of *CmARF2a*, which is located on a scaffold (Figure 3A). Chromosome 1, the longest chromosome, harbors four *CmARF* members: *CmARF1*, *CmARF8*, *CmARF9*, and *CmARF5a*. Chromosomes 2, 5, 6, 11, and 12 each contain two *CmARF* genes. In contrast, Chromosomes 3, 4, and 9 each support a single *CmARF* member, while Chromosomes 7, 8, and 10 have no *CmARF* genes. Notably, there appears to be no correlation between the length of a chromosome and the presence of *CmARF* members. Gene duplication constitutes a pivotal mechanism underpinning the evolution of gene families and the diversification of gene functions [49]. Utilizing collinear analysis within the *C. mollissima* genome, and extending this analysis to include homologous collinear dot plots, we previously classified the duplication types of *CmARF* genes (Figure 3B,C) [28,34]. Our findings reveal that dispersed duplication accounts for approximately 66.67% (12 genes) of all *CmARF* genes, suggesting this duplication type plays a significant role in the expansion of the CmARF gene family. Additionally, *CmARF10*, *CmARF16a*, and *CmARF16b* are derived from the core eudicot common hexaploidization (ECH) event [33]. Segmental duplication is observed in *CmARF2a* and *CmARF2b*, while *CmARF8* is associated with proximal duplication. Furthermore, the non-synonymous (Ka) to synonymous (Ks) substitution rate ratios for the gene pairs within the *CmARF* family are all below 1, indicating that these genes have been subject to purifying selection throughout their evolutionary history (Table S4).

### 3.4. Collinear Analysis of ARFs in Several Plant Species

**Figure 4:**
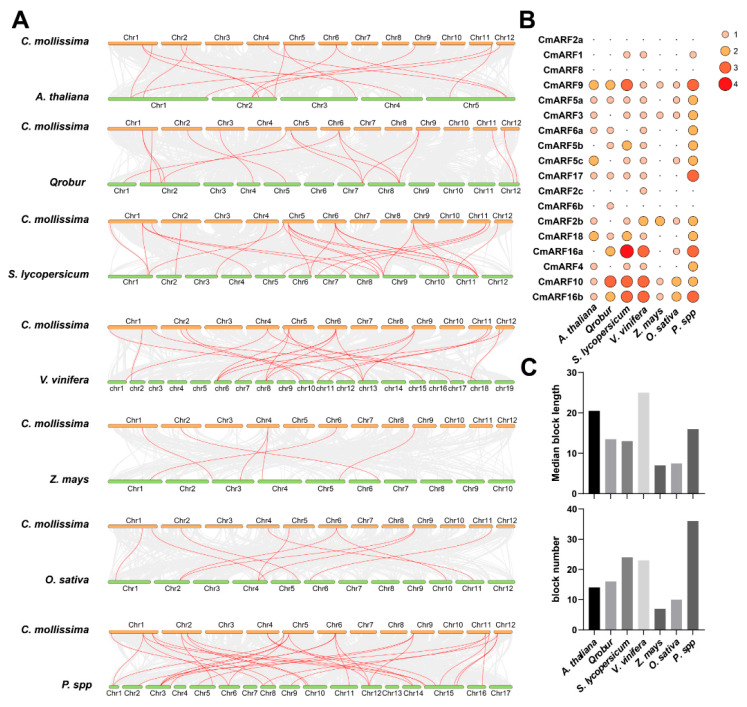
Collinear analyses between the *CmARF* genes and genes in seven representative plant species (*Q. robur*, *P. bretschneideri*, *V. vinifera*, *A. thaliana*, *S. lycopersicum*, *O. sativa*, and *Z. mays*). (**A**) The dual collinear plot between *C. mollissima* and seven representative plant species. Gray lines in the background indicate collinear blocks within *C. mollissima* and other plant genomes, while red lines highlight collinear *ARF* gene pairs. (**B**) The orthologous gene pair number between *C. mollissima* and other seven plant genomes. (**C**) The block number and median block length between *C. mollissima* and other seven plant genomes.

A gene family is generally defined as a collection of genes descended from a common ancestor, comprising two or more copies of a gene that have arisen through gene duplication [50]. In this study, seven representative species were selected to examine their collinear with the *C. mollissima* genome, building upon our previous research [28]. The analysis identified varying numbers of orthologous gene pairs containing *CmARFs* distributed across the collinear regions of *C. mollissima* and several other species: *A. thaliana* (14), *Q. robur* (15), *S. lycopersicum* (24), *V. vinifera* (22), *Z. mays* (6), *O. sativa* (10), and *P. bretschneideri* (31) (Figure 4A,B; Tables S5–S11). Additionally, the collinear regions between *C. mollissima* and these species contained 11, 10, 13, 15, 5, 8, and 14 *CmARF* genes, respectively (Tables S5–S11). It is evident that additional orthologous gene pairs have been retained between *C. mollissima* and dicotyledonous plants relative to monocotyledonous plants, suggesting independent duplication events in the ARF gene family during the evolution of dicotyledonous plants. Notably, *CmARF10* is associated with three orthologous gene pairs in *Q. robur*, *S. lycopersicum*, and *V. vinifera*, which indicates a higher preservation of homologous genes of *CmARF* in these species (Figure 4A,B; Tables S5–S11). Similarly, both *CmARF16a* and *CmARF16b* are linked to at least three orthologous genes in the genomes of *V. vinifera*, *S. lycopersicum*, and *P. bretschneideri*, respectively. The gene dosage hypothesis posits that genes integral to complex regulatory networks are more likely to be conserved through evolution due to their sensitivity to changes in gene dosage (i.e., gene copy number), suggesting a crucial role for *CmARF10*, *CmARF16a*, and *CmARF16b* in the evolutionary trajectory of the CmARF gene family [51,52]. A diverse array of collinear blocks containing *CmARFs* was observed between *C. mollissima* and *A. thaliana* (14), *Q. robur* (16), *S. lycopersicum* (24), *V. vinifera* (23), *Z. mays* (7), *O. sativa* (10), and *P. bretschneideri* (36), with median block lengths of 20.5, 13.5, 13.0, 25.0, 7.0, 7.5, and 16, respectively (Figure 4C; Tables S12–S18). These findings illuminate the evolutionary relationships among these species and underscore the retention of the ARF gene family within collinear regions.

### 3.5. Cis-Acting Elements and TFs Regulatory Network

**Figure 5:**
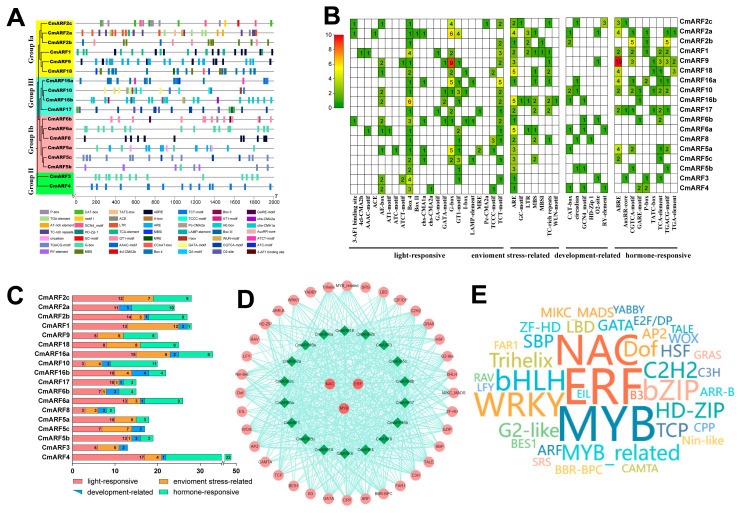
Prediction of *cis*-acting elements and transcription factor regulatory network analysis of *CmARFs*. (**A**) *Cis*-acting elements in the promoters of *CmARFs*. Various color symbols present different elements, and their position in the figure indicates their relative position on the promoter. (**B**) The number of various *cis*-acting elements in the promoters of each *CmARFs*. (**C**) The relative proportions of different *cis*-acting elements in the promoters of *CmARFs* are indicated in the chart. (**D**) The putative TFs regulatory network analysis of *CmARF* genes. (**E**) Word-cloud of predicted TFs interacting with *CmARF* genes. The font size is positively correlated with the number of corresponding TFs.

Promoters are DNA regulatory sequences situated upstream of genes [53]. Located within the promoter region, specific sequences initiate transcription by binding to RNA polymerase and TFs, thereby regulating the spatiotemporal specificity and intensity of gene expression [54]. In the promoters of 18 *CmARFs*, a total of 417 *cis*-acting elements were predicted (Figure 5A–C; Table S19). Notably, a substantial assortment of *cis*-acting elements associated with development and hormones was found across nearly all *CmARFs*’ promoter regions. These include the auxin-responsive element (TGA-element), gibberellin-responsive element (P-box), MeJA-responsive *cis*-acting regulatory element (TGACG-motif), salicylic acid responsive *cis*-acting element (TCA-element), and a *cis*-regulatory element involved in endosperm expression, suggesting a broad involvement of *CmARFs* in the growth and development of *C. mollissima*. Additionally, other *cis*-acting elements related to light responses and environmental pressures were identified, including the light-responsive element (GT1-motif), MYB binding site involved in drought response (MBS), elements involved in defense and stress responses (TC-rich repeats), wound responsive element (WUN motif), and *cis*-acting element involved in low-temperature responsiveness (LTR). These findings align with the extensively documented role of ARF genes in plant growth, development, and environmental stress response [2]. Moreover, TFs potentially acting on the CmARFs were also predicted (Figure 5D,E; Table S20). A total of 308 TFs from 39 gene families were identified as likely regulators of CmARFs expression. Notable among these TFs were ERF (39), MYB (38), NAC (31), and WRKY (23), indicating their significant roles in the regulation of *CmARFs* expression. Additionally, certain TF families were represented by only one member potentially influencing CmARF gene expression, such as GRAS, TALE, CAMTA, and STS.

### 3.6. Protein Interaction Network and GO/KEGG Enrichment Analysis

**Figure 6:**
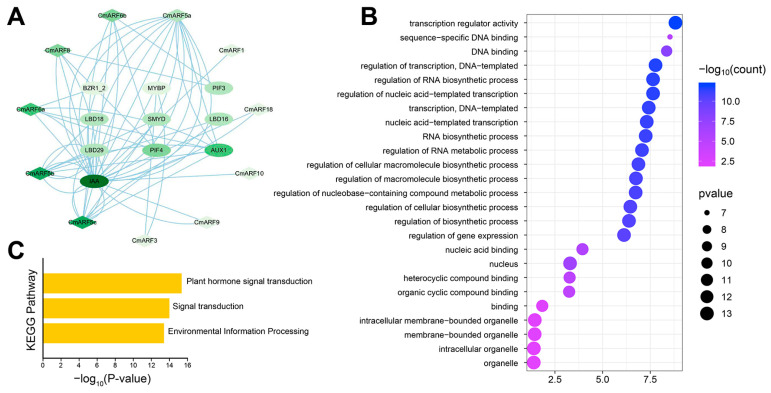
Protein interaction network and GO/KEGG analysis of *CmARFs*. (**A**) Protein interaction network of CmARF proteins. (**B**) GO function enrichment analysis of *CmARF* genes. (**C**) KEGG function enrichment analysis of *CmARF* genes.

The secondary and tertiary structures of proteins encoded by the CmARF gene were predicted to characterize these proteins. The secondary structure predominantly consists of random coils, ranging from 70.6% to 79.6%, alpha helices from 10.8% to 17.8%, and extended strands from 8.2% to 13.2%, with an absence of beta-turns (Table S21). The tertiary structure predictions indicate that CmARF proteins within the same subfamily exhibit similar conformations (Figure S1). Additionally, the interaction network of CmARF proteins was inferred using the String online tool [38] (Figure 6A). Consistent with expectations, IAA proteins were identified at the core of this network. This centrality is attributable to the ability of ARF proteins, which are TFs, to recognize and bind IAA, thereby regulating the expression of genes responsive to growth hormones [2]. Notably, CmARF5b and CmARF5c occupy central positions within this protein-interaction network, suggesting their pivotal roles in the regulation of growth and development in *C. mollissima*. Furthermore, Gene Ontology (GO) and Kyoto Encyclopedia of Genes and Genomes (KEGG) enrichment analyses revealed that *CmARF* genes are primarily enriched in pathways related to plant hormone signal transduction, transcriptional regulator activity, and the regulation of RNA biosynthetic processes (Figure 6B,C). These findings align with the known role of ARF as a key component of the auxin signaling pathway [55].

### 3.7. Phenotypic Changes During the Development of C. mollissima Seed Kernels

**Figure 7:**
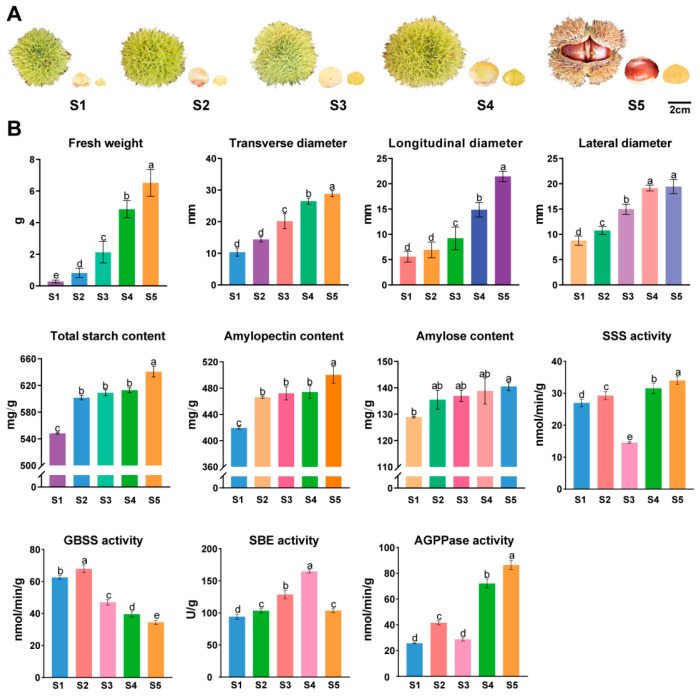
Dynamic changes in morphology and physiological indicators of different developmental stages of ‘Xinglong No. 4’ *C. mollissima*. (**A**) The thorns bract, seeds, and seed kernels of ‘Xinglong No. 4’ *C. mollissima* at different developmental stages. (**B**) The fresh weight, longitudinal diameter, transverse diameter, lateral diameter, total starch content, amylopectin content, amylose content, SSS, GBSS, SBE, and AGGPase activity of seed kernels of ‘Xinglong No. 4’ *C. mollissima* at different developmental stages. The small letters indicate the significance of differences between physiological indicators in five stages.

The variety ‘Xinglong No. 4’ of *C. mollissima* exhibited significant morphological changes in both the thorn bracts and seed kernels during development, primarily characterized by an increase in size. The thorn bracts opened 100 days post-flowering, denoting the onset of maturity (Figure 7A). An essential trait under investigation was the size of the seed kernels. Initially, at stage S1, the fresh weight of the seed kernel was a mere 0.28 g; subsequently, it progressively increased, reaching a peak of 6.52 g at stage S5 (Figure 7B). Similarly, the dimensions of the seed kernels expanded over time, with their longitudinal, transverse, and lateral diameters starting at 5.60 mm, 10.40 mm, and 8.76 mm, respectively, at stage S1 and culminating at 21.45 mm, 28.85 mm, and 19.44 mm at maturity (stage S5). The growth rate of the seed kernel size was notably accelerated between stages S3 and S4. Starch constitutes the primary nutrient in the seed kernels of *C. mollissima* [56]. At the initial stage S1, the total starch content was recorded at 548.25 mg/g, representing 54.83% of the dry weight of the seed kernels (Figure 7B). This starch content increased, reaching its highest at 640.74 mg/g at stage S5. Within the starch composition, amylopectin was predominant, while amylose was less abundant. The content of amylopectin and amylose initially was 419.38 mg/g and 128.87 mg/g, respectively, at stage S1, escalating to their respective maxima of 500.25 mg/g and 140.49 mg/g at stage S5. Key enzymes implicated in starch biosynthesis in *C. mollissima*, such as granule-bound starch synthase (GBSS), starch branching enzyme (SBE), soluble starch synthase (SSS), and adenosine diphosphate glucose pyrophosphatase (AGPPase), have been identified [29,30,56]. GBSS activity was significantly higher during the initial stages (S1 and S2) and thereafter decreased to its lowest level at stage S5 (Figure 7B). Conversely, the activities of AGPPase and SBE generally increased with development, though SBE activity notably declined at stage S5. Statistical analysis confirmed that SSS activity was significantly lower at stage S3 compared to S1, S2, S4, and S5, before increasing as development progressed.

### 3.8. RNA-Sequencing and DEG Identification

**Figure 8:**
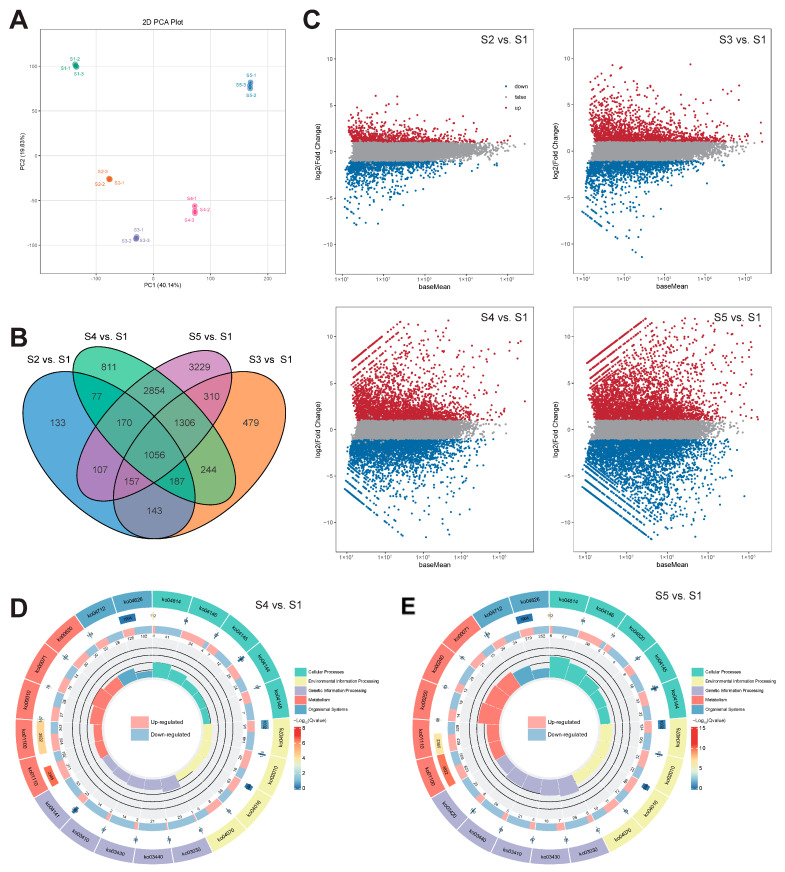
Transcriptome analysis results. (**A**) PCA analysis of transcriptome data. (**B**) DEGs distribution in four comparisons at different developmental stages. (**C**) DEGs of S2 vs. S1, S3 vs. S1, S4 vs. S1, and S5 vs. S1, respectively. (**D**) KEGG enrichment of DEGs in S4 vs. S1. (**E**) KEGG enrichment analysis of DEGs in S5 vs. S1.

mRNA was extracted from the seed kernels of ‘Xinglong No. 4’ *C. mollissima* across five developmental stages, leading to the construction of 15 cDNA libraries (three replicates per stage). Transcriptome sequencing yielded a total of 878.66 Mb of clean reads, characterized by high base quality (Q20 ≥ 98.01%, Q30 ≥ 94.23%) and a consistent GC content ranging between 43.62% and 44.32% (Table S22). The alignment rates to the reference genome varied from 88.82% to 90.15% (Table S23). PCA distinctly differentiated the RNA-seq data from various developmental stages, highlighting significant differences in gene expression profiles among the groups (Figure 8A).

To investigate the underlying mechanisms of kernel development and starch biosynthesis in *C. mollissima*, DEGs were identified across various developmental stages using stringent criteria: an absolute log2 FC of at least 2 and an FDR below 0.05. Relative to stage S1, there was a significant down-regulation in the expression of 1191, 2101, 3480, and 4712 genes at stages S2, S3, S4, and S5, respectively. Concurrently, 840, 1782, 3226, and 4478 genes demonstrated significant up-regulation at these stages (Figure 8B,C). Notably, the number of DEGs increased progressively as the *C. mollissima* kernels matured, with the number of down-regulated genes consistently surpassing that of up-regulated genes. The predominance of down-regulated genes during development may reflect a coordinated shutdown of specific metabolic and biosynthetic pathways as cells transition to more specialized states [57]. Enrichment analyses via KEGG and GO pathways revealed that these DEGs were predominantly associated with the biosynthesis of secondary metabolites, plant hormone signal transduction, starch and sucrose metabolism, and the MAPK signaling pathway (Figure 8D; Figures S2 and S3).

**Figure 9:**
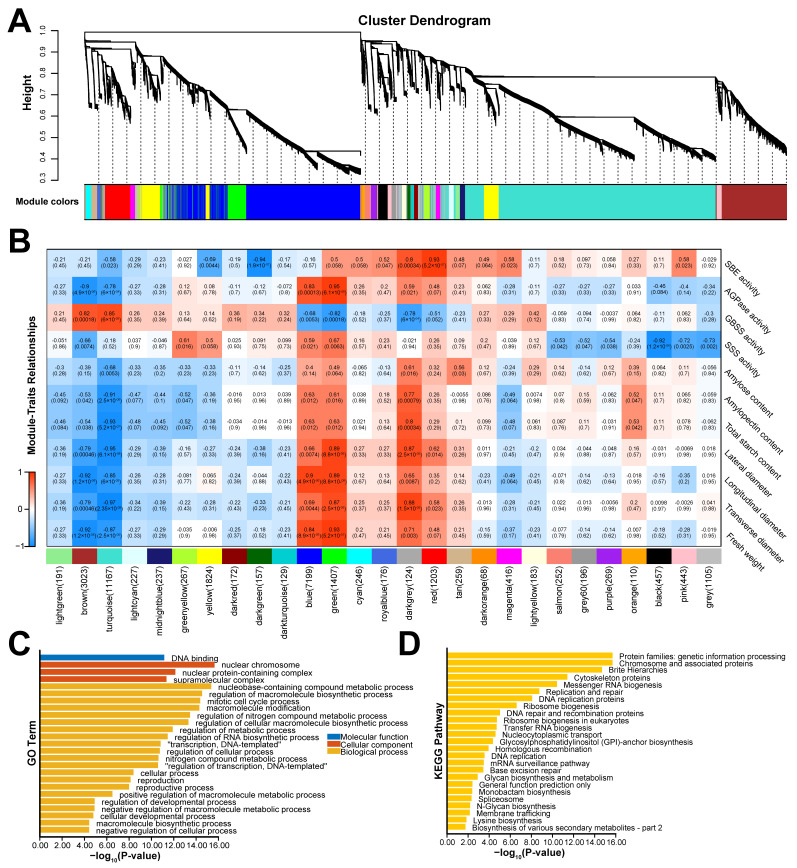
WGCNA and functional enrichment analysis of the turquoise module. (**A**) The clustering dendrogram of genes identifying the WGCNA modules. (**B**) The correlation of the identified modules with the phenotypic traits at different developmental stages. (**C**) GO enrichment analysis of the turquoise modules. (**D**) KEGG enrichment analysis of the turquoise modules.

WGCNA was employed to identify core genes critical to the development of *C. mollissima* seed kernels, utilizing RNA-seq data from five developmental stages alongside corresponding phenotypic data (Figure 9A). Analysis of the 27 identified modules revealed varied correlations with different phenotypic aspects of the seed kernel (Figure 9B). Noteworthy were the significant correlations observed in some modules with kernel size and starch content. For instance, the turquoise module displayed significant negative correlations with fresh weight (*r* = −0.87, *p* = 2.5 × 10^−5^), longitudinal diameter (*r* = −0.97, *p* = 2.3 × 10^−9^), transverse diameter (*r* = −0.85, *p* = 6 × 10^−5^), lateral diameter (*r* = −0.95, *p* = 6.1 × 10^−8^), total starch (*r* = −0.93, *p* = 5.2 × 10^−7^), and amylopectin content (*r* = −0.91, *p* = 2.5 × 10^−6^). Conversely, the green module exhibited significant positive correlations with fresh weight (*r* = 0.93, *p* = 5.2 × 10^−7^), longitudinal diameter (*r* = 0.89, *p* = 8.8 × 10^−6^), transverse diameter (*r* = 0.87, *p* = 2.5 × 10^−5^), and lateral diameter (*r* = 0.89, *p* = 8.8 × 10^−6^). These findings suggest that the gene sets and their expression regulatory networks within these modules are intricately linked to kernel development and starch biosynthesis in *C. mollissima*. Intriguingly, ten of the eighteen *CmARF* genes were located within the turquoise module, implying a potential role in kernel development and starch accumulation (Table S24). GO and KEGG functional enrichment analyses of the turquoise module indicated significant enrichment in cellular developmental processes, biosynthesis of various secondary metabolites, and positive regulation of macromolecule metabolic processes, underscoring the module’s relevance to *C. mollissima* seed kernel development (Figure 9C,D).

### 3.9. Correlation Between CmARF Genes and Phenotypic Indicators, and RT-qPCR Validation

**Figure 10:**
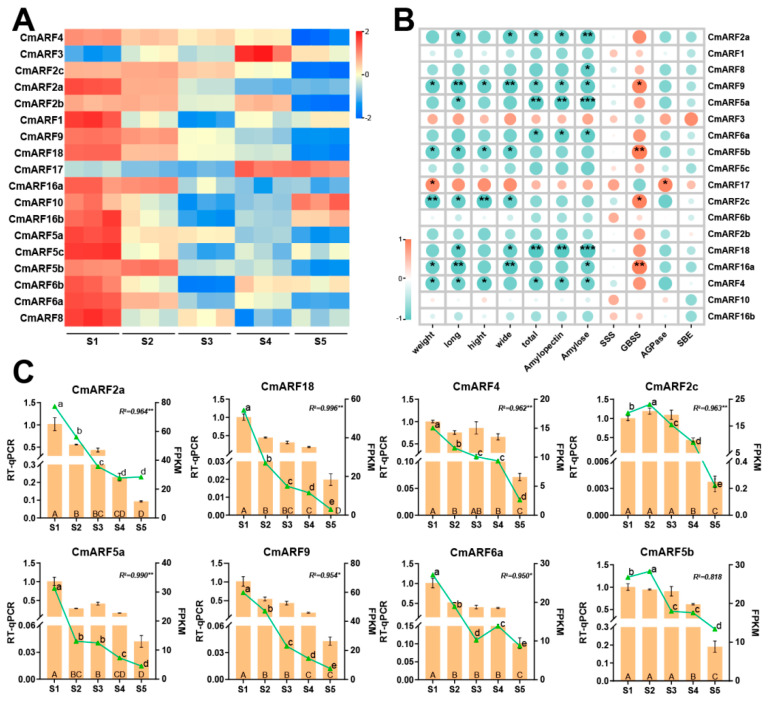
The expression profiles of *CmARF* genes, RT-qPCR at different developmental stages of ‘Xinglong No. 4’ *C. mollissima* seed kernels, and their correlation with phenotypic indicators. (**A**) The expression profiles of *CmARFs* at different developmental stages of ‘Xinglong No. 4’ *C. mollissima* seed kernels. (**B**) The correlation between phenotypic indicators and *CmARFs* expression levels at different developmental stages of ‘Xinglong No. 4’ *C. mollissima* seed kernels. ***, ** and * denote genes and physiological indicators exhibiting significant differences (*p* < 0.001, *p* < 0.01, *p* < 0.05 respectively) (**C**) RT-qPCR analysis of *CmARFs* in *C. mollissima* at different developmental stages of ‘Xinglong No. 4’ *C. mollissima* seed kernels. The lowercase letters indicate the significance of differences between FPKM of transcriptome data from five stages, while uppercase letters indicate the significance of differences between RT-qPCR results from five stages.

The significant roles of the ARF gene family in plant growth and development have been extensively documented [2,24,25,26]. Notably, certain members of the *CmARF* family exhibit pronounced fluctuations in expression during the development of *C. mollissima* seed kernels (Figure 10A). For instance, the expression levels of *CmARF9* and *CmARF5a* were elevated at the S1 stage, with FPKM values of 59.88 and 31.32, respectively. These levels subsequently declined markedly, reaching their nadir at the maturation stage (S5) with values of only 7.58 and 4.52, respectively (Figure 10A). Additionally, an analysis was conducted to assess the correlation between the expression levels of *CmARF* genes and associated phenotypic indicators, aiming to elucidate the potential roles of *CmARF* genes in seed kernel development (Figure 10B). Genes that exhibited significant associations with seed kernel size and starch content were of particular interest. This focus stems from the observation that seed kernel size represents the most conspicuous change during development, and starch constitutes the primary nutrient in seed kernels, typically comprising about 50% of their dry weight [58,59]. Notably, as detailed in Table S25, the expression of *CmARF5a* (*r* = −9.78, *p* < 0.01) and *CmARF18* (*r* = −9.81, *p* < 0.01) were highly significantly negatively correlated with total starch content. Conversely, *CmARF9*, *CmARF5b*, *CmARF2c*, and *CmARF4* demonstrated significant negative correlations with traits related to seed kernel size (fresh weight, longitudinal diameter, transverse diameter, and measured diameter), indicating their associations with seed kernel development. Selected *CmARF* genes, which displayed substantial expression differences during the development phases of *C. mollissima* seed kernels, were chosen for RT-qPCR experiments to validate the transcriptome data (Figure 10C). The outcomes of these RT-qPCR experiments exhibited robust consistency and correlation with the RNA-seq data, thereby confirming the dynamic expression of *CmARF* genes throughout seed kernel development.

## 4. Discussion

### 4.1. Identification and Molecular Characteristics of ARF Genes in C. mollissima

In this study, 18 CmARF genes were identified in the *C. mollissima* genome. The encoded proteins were all predicted to be hydrophilic and unstable, a common characteristic of ARF proteins across plant species [2,11,13,15,26]. These properties could potentially explain their generally low expression in heterologous systems and may present challenges for functional validation in *C. mollissima* [60]. Phylogenetic analysis classified the *CmARF* genes into four distinct groups, consistent with the categorization in other plants [61] (Figure 1). All CmARF proteins possess the conserved B3 DNA-binding domain (DBD) and Auxin Responsive Domain (ARD), which are considered essential for their functional [62]. Gene structure analysis revealed that members within the same phylogenetic group generally share similar exon-intron patterns (Figure 2). Notably, groups Ia and Ib contained more introns (13–15) than group III (2–8), a structural variation that might be relevant to gene evolution and expression regulation [63,64,65]. Furthermore, CmARF proteins within the same group exhibited similar three-dimensional structures (Figure S1), implying potential functional conservation. This structural similarity could aid in predicting their roles by analogy with characterized ARF proteins from model species like *A. thaliana* [66].

### 4.2. Evolution and Expansion of CmARFs

The number of ARF genes in *C. mollissima* (18) is similar to that in *V. vinifera* (19) [12], *P. mume* (17) [13], and *P. persica* (17) [14]. Collinear analysis suggested that *C. mollissima* exhibited closer evolutionary relationships with dicotyledonous plants than monocots (Figure 4, Tables S5–S18). Some CmARF members, such as *CmARF16a* and *CmARF16b*, possessed multiple orthologous genes in several dicot species, which might indicate potential functional importance or diversification. Conversely, *CmARF2a* and *CmARF8* lacked clear orthologous genes in the seven surveyed plant genomes, hinting at potential functional specificity in *C. mollissima*. Analysis of duplication events indicated that dispersed duplication was the primary mechanism (66.67% of genes) for the expansion of the CmARF gene family (Figure 3), aligning with its recognized role as a major driver of gene family evolution in plants [67,68].

### 4.3. Promoter Analysis and Protein Interaction Network Analysis

*Cis*-acting element analysis revealed that the promoter regions of *CmARF16a* and *CmARF16b* were particularly enriched with elements related to seed development, including those involved in endosperm and meristem expression, and gibberellin response (Figure 5; Table S19). This finding is consistent with their potential role in seed development, especially given that their *A. thaliana* orthologous genes, *AtARF16*, inhibits embryonic axis elongation [69]. Protein interaction network analysis predicted *CmARF5b* and *CmARF5c* as central hubs (Figure 6A), analogous to the critical role of *AtARF5* in embryonic development [2]. Additionally, the promoter of *CmARF10* was predicted to be regulated by multiple TFs (e.g., MYB, NAC, TCP) (Figure 5D,E), suggesting its involvement in complex regulatory networks, potentially in processes like lateral root formation as seen with *AtARF10* [70].

### 4.4. Potential Functional Analysis of CmARFs During C. mollissima Seed Kernel Development

The role of *ARF* genes in fruit and seed development is well-established [21,22,70]. In this study, we investigated their involvement in the development of *C. mollissima* seed kernels, which are notable for their high starch content [29,30]. We observed significant negative correlations between the expression of several *CmARF* genes (e.g., *CmARF9*, *CmARF5b*, *CmARF2c*) and seed kernel size (Figure 10), which aligns with the known functions of *ARFs* like *SlARF7* and *AtARF8* in limiting organ growth [17,71].

Our most intriguing finding pertains to *CmARF5a* and *CmARF18*. Their expression showed highly significant negative correlations with total starch, amylopectin, and amylose content. However, a critical and novel observation was the lack of significant correlation between their expression and the key genes encoding starch biosynthetic enzymes (SSS, GBSS, AGPase, SBE). The absence of a direct correlation with the expression of core starch biosynthetic genes suggests that *CmARF5a* and *CmARF18* may act as indirect repressors, potentially modulating upstream processes like carbon partitioning or photosynthetic efficiency. This proposed indirect mechanism represents a testable hypothesis for future research.

We hypothesize that these TFs might modulate upstream processes such as photosynthesis or carbohydrate transport. This interpretation is supported by: (1) the abundance of photosynthesis-related cis-elements in their promoter regions (Figure 5), and (2) their membership in the WGCNA turquoise module, which is significantly correlated with starch content and enriched in pathways like “biosynthesis of secondary metabolites” and “positive regulation of macromolecule metabolic processes,” but not directly in starch biosynthesis itself (Figure 9). The role of *AtARF5* as a transcriptional activator in complex developmental processes provides a plausible precedent for *CmARF5a* acting through multifaceted pathways [70,72]. The pronounced decrease in *CmARF5a* and *CmARF18* expression during seed kernel maturation, as confirmed by RT-qPCR, is consistent with a potential role as repressors of starch accumulation.

In conclusion, this study provides the first systematic characterization of the CmARF gene family in *C. mollissima*. Beyond cataloging these genes, our integrated analysis points to a potential novel regulatory paradigm where specific *CmARF* genes (*CmARF5a* and *CmARF18*) may control starch accumulation in perennial woody crops through indirect mechanisms, distinct from the direct transcriptional regulation of biosynthetic genes often reported in other systems. These findings propose a framework for future functional studies aimed at validating these mechanisms and improving starch-related traits in *C. mollissima*.

### 4.5. Limitations and Future Perspectives

While this study provides comprehensive insights into the CmARF gene family, several limitations should be considered when interpreting the results. First, the associations between *CmARF* genes expressions and phenotypic traits are primarily correlative. Although these correlations provide valuable clues about potential gene functions, they do not establish causal relationships. Second, functional validation of the proposed roles of key *CmARF* genes, particularly *CmARF5a* and *CmARF18*, through transgenic experiments in model plants or *C. mollissima* itself is ultimately required to confirm their biological functions. Such experiments remain challenging in perennial woody crops like *C. mollissima* due to long life cycles and transformation difficulties, but they represent a crucial next step. Third, our study focused on a single elite cultivar, ‘Xinglong No. 4’. Consequently, the generalizability of the observed expression patterns and correlations to other *C. mollissima* cultivars with different genetic backgrounds requires further investigation. Future work should include multiple cultivars to assess the broader applicability of these findings. Furthermore, the identification of gene family members based on domain and motif structure, while standard, may exclude atypical members; future functional studies could clarify the status of such genes.

Furthermore, the observed decrease in GBSS activity during later developmental stages, which coincided with a marked increase in total starch content (Figure 7B), may initially seem paradoxical. However, this pattern can be explained by the concurrent dynamic of a significantly increasing amylopectin-to-amylose ratio. As GBSS is primarily responsible for amylose synthesis, its reduced activity likely contributes to a relative enrichment of amylopectin, the major component of starch. The substantial increase in the activities of AGPase and SBE, key enzymes for amylopectin synthesis and branching, during these stages (Figure 7B) supports this interpretation and accounts for the net accumulation of total starch.

Despite these limitations, the current study establishes a solid foundation and identifies specific candidate genes for such functional studies, which will be vital for elucidating the precise regulatory mechanisms and for genetic improvement of *C. mollissima*.

## 5. Conclusions

In this study, a total of 18 ARF gene family members were identified in the *C. mollissima* genome. Phylogenetic analysis classified them into four distinct subfamilies. Further examination revealed that all *CmARF* genes contain typical ARF conserved domains and exhibit high conservation in gene structure and motif composition. Integrated analyses involving collinearity assessment, promoter *cis*-acting element screening, transcription factor binding prediction, functional enrichment, and protein interaction networks collectively suggested potential roles of *CmARF* genes in *C. mollissima* growth, development, and starch accumulation. Transcriptomic profiling and RT-qPCR validation demonstrated that all 18 *CmARF* genes were differentially expressed across five developmental stages of *C. mollissima* seed kernels, showing significant spatiotemporal specificity. Among these, *CmARF5a* and *CmARF18* were identified as key regulators potentially involved in seed kernels development and starch metabolism. However, the precise molecular mechanisms through which these genes mediate seed kernels development warrant further investigation. This study provides a foundation for functional characterization of ARF gene families in woody plants and offers theoretical insights and candidate genes for future research on the regulation of seed development and starch metabolism in *C. mollissima*.

## Data Availability

The original contributions presented in this study are included in the article and Supplementary Materials. The RNA-seq data generated by this study were uploaded to NCBI and can be accessed through the BioProject login number PRJNA1268182.

## Supplementary Materials

The following supporting information can be downloaded at: https://www.mdpi.com/article/10.3390/biology14101460/s1, Figure S1: The predicted three-dimensional structure of CmARF proteins; Figure S2: KEGG enrichment of DEGs in S2 vs. S1, S3 vs. S1; Figure S3: GO enrichment of DEGs in S2 vs. S1, S3 vs. S1, S4 vs. S1, and S5 vs. S1; Table S1: ARF genes list from *C. mollissima*, *A. thaliana*, *O. sativa*; Table S2: Primers used for RT-qPCR analysis. Table S3: Accession numbers and characteristics of 18 *CmARF* genes in *Castanea mollissima*; Table S4: The duplication model and calculation of Ka and Ks ratios of duplicated *CmARF* gene pairs; Table S5: The orthologous gene pairs containing *CmARF* in the cross of chestnut and *Arabidopsis*; Table S6: The orthologous gene pairs containing *CmARF* in the cross of chestnut and grape; Table S7: The orthologous gene pairs containing *CmARF* in the cross of chestnut and oak; Table S8: The orthologous gene pairs containing *CmARF* in the cross of chestnut and rice; Table S9: The orthologous gene pairs containing *CmARF* in the cross of chestnut and tomato; Table S10: The orthologous gene pairs containing *CmARF* in the cross of chestnut and pear; Table S11: The orthologous gene pairs containing *CmARF* in the cross of chestnut and maize; Table S12: The collinear blocks containing *CmARF* identified between chestnut and grape; Table S13: The collinear blocks containing *CmARF* identified between chestnut and *Arabidopsis thaliana*; Table S14: The collinear blocks containing *CmARF* identified between chestnut and oak; Table S15: The collinear blocks containing *CmARF* identified between chestnut and rice; Table S16: The collinear blocks containing *CmARF* identified between chestnut and tomato; Table S17: The collinear blocks containing *CmARF* identified between chestnut and pear; Table S18: The collinear blocks containing *CmARF* identified between chestnut and maize; Table S19: Information of cis-acting elements predicted from 2000 bp upstream regions of 21 *CmARF* genes; Table S20: Predictive transcription factor (TFs) analysis of *CmARF* genes; Table S21: CmARF protein secondary structure; Table S22: Summary of transcriptome sequencing; Table S23: Mapping rate range information statistics with reference genome; Table S24: The distribution of *CmARF* genes in color modules in WGCNA; Table S25: Correlation analysis between CmARF gene expression level and phenotype indicators.
