# Supplementary figures and images for: Comprehensive Analysis of the ARF Gene Family Reveals Their Roles in Chinese Chestnut (Castanea mollissima) Seed Kernel Development
Source: Biology (Basel). 2025 Oct 21;14(10):1460. doi: 10.3390/biology14101460 (PMC12561081; doi:10.3390/biology14101460)

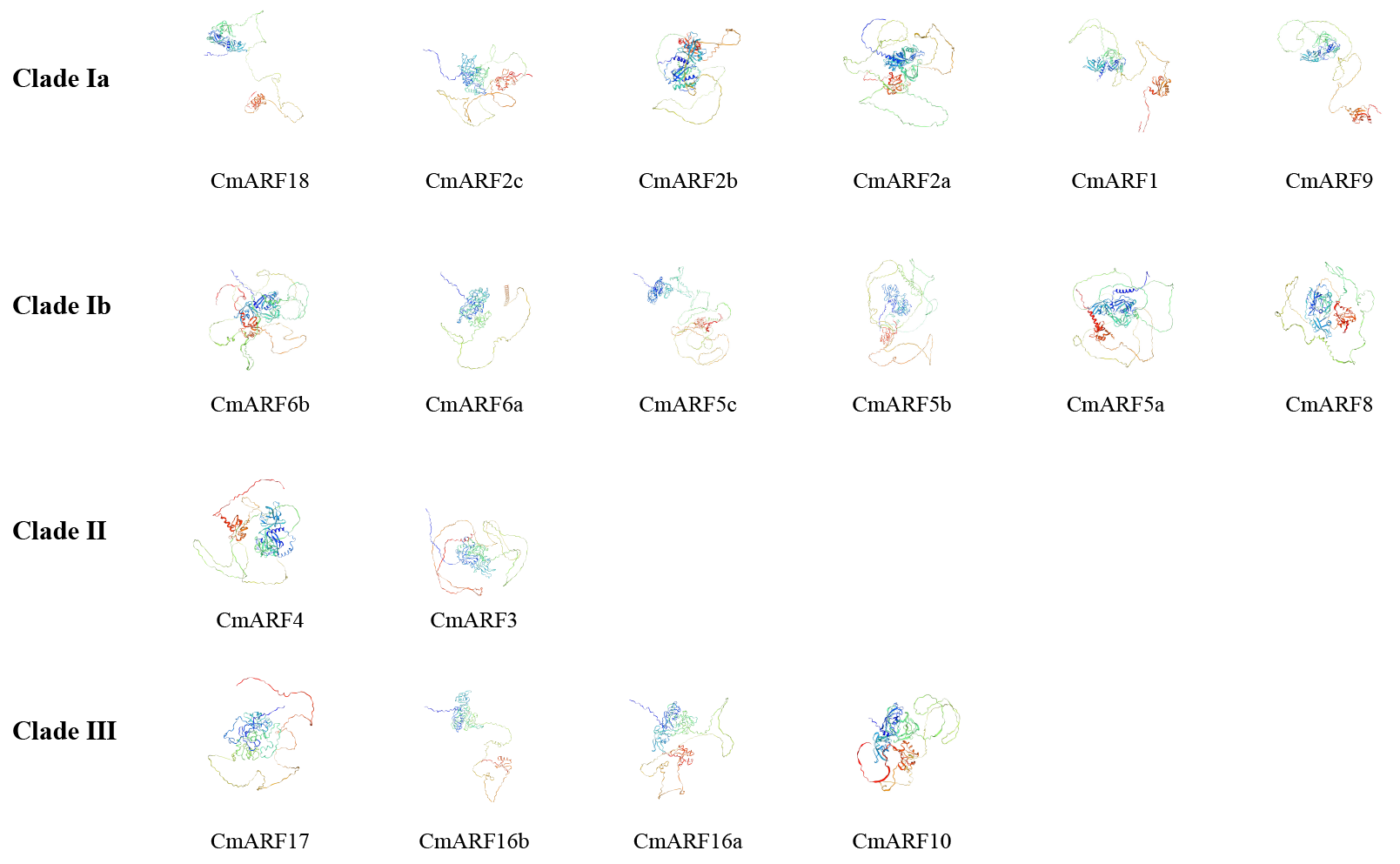

Supplement: Supplementary file 1 [file biology-14-01460-s001.zip › Figure S1.tif]

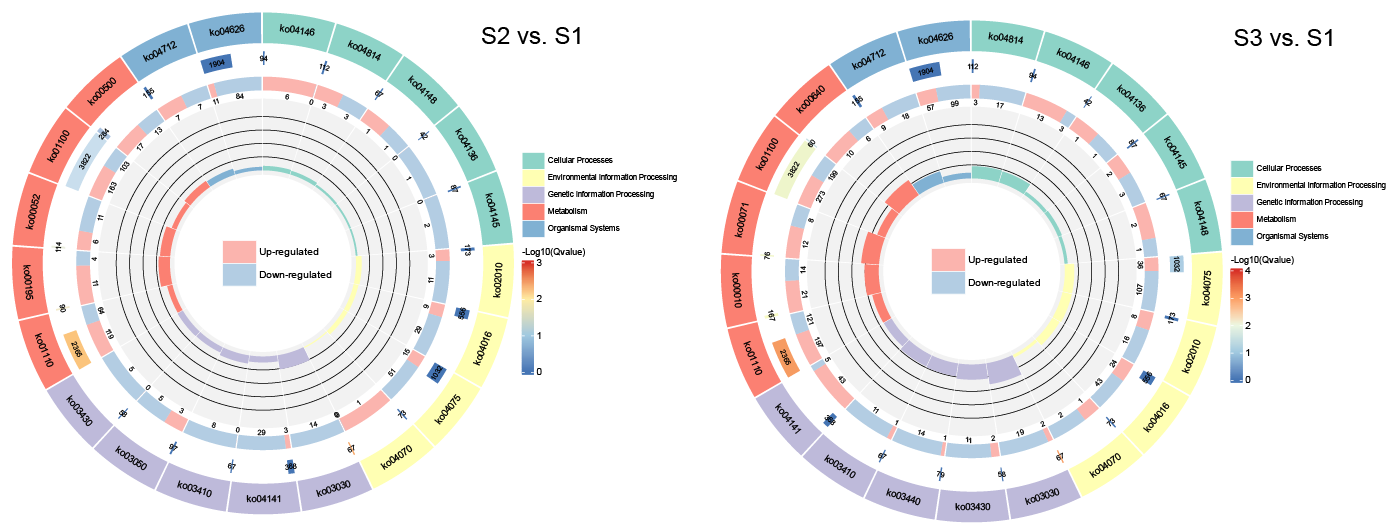

Supplement: Supplementary file 1 [file biology-14-01460-s001.zip › Figure S2.tif]

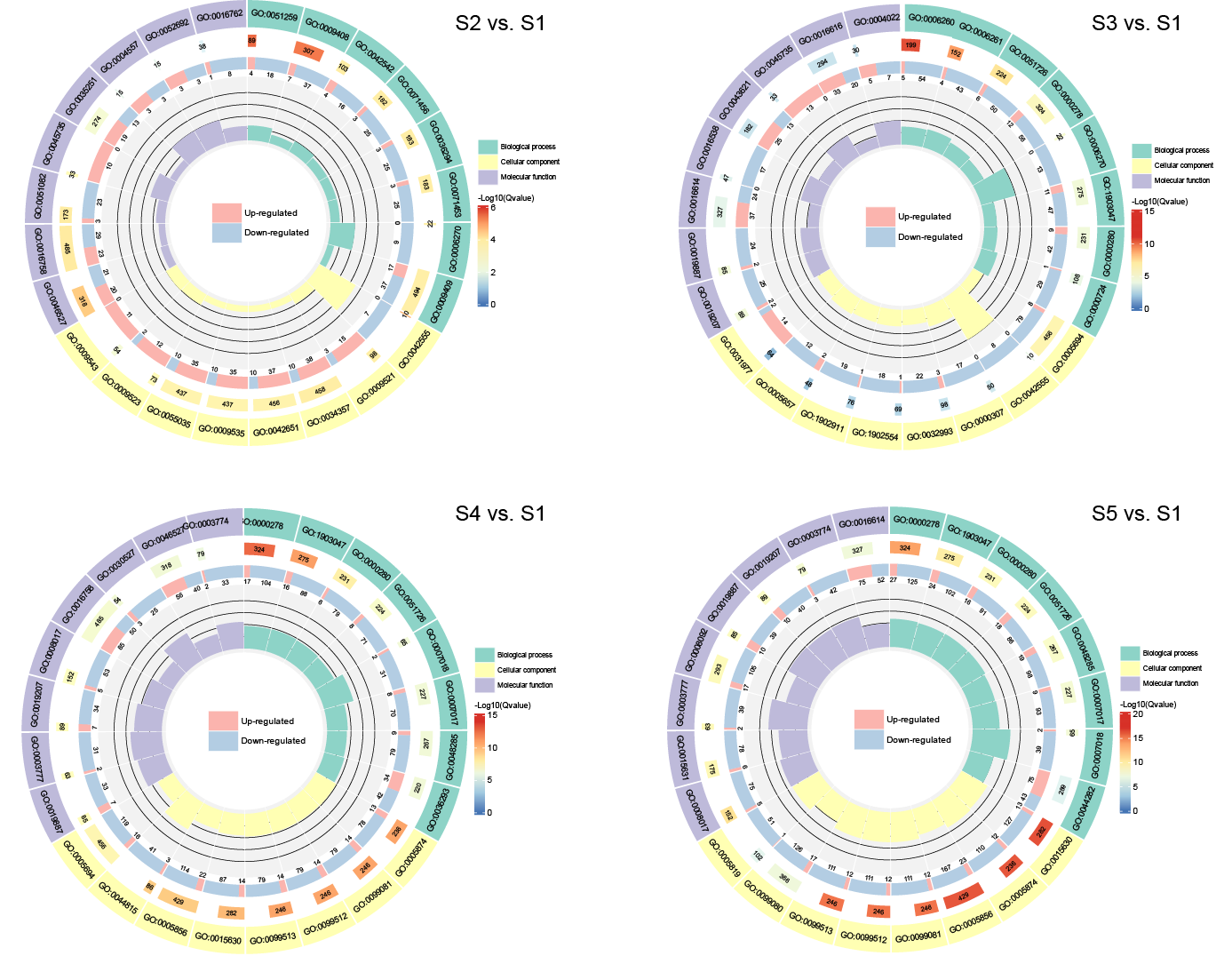

Supplement: Supplementary file 1 [file biology-14-01460-s001.zip › Figure S3.tif]
